# The value of multi-parameter radiomics combined with imaging features in predicting the therapeutic efficacy of HIFU treatment for uterine fibroids

**DOI:** 10.3389/fonc.2024.1499387

**Published:** 2024-11-20

**Authors:** Li Shen, Xiao Huang, YuYao Liu, QingXue Li, ShanWei Bai, Fang Wang, Quan Yang

**Affiliations:** ^1^ Department of Radiology, The Affiliated Yongchuan Hospital of Chongqing Medical University, Chongqing, China; ^2^ Department of Radiology, The Second Affiliated Hospital of Chongqing Medical University, Chongqing, China; ^3^ Department of Research and Development, Shanghai United Imaging Intelligent Co., Ltd, Shanghai, China

**Keywords:** high-intensity focused ultrasound, uterine fibroids, magnetic resonance imaging, radiomics, therapeutic efficacy

## Abstract

**Objectives:**

To evaluate the effectiveness of high-intensity focused ultrasound (HIFU) therapy for treating uterine fibroids by utilizing multi-sequence magnetic resonance imaging radiomic models.

**Methods:**

One hundred and fifty patients in our hospital were randomly divided into a training cohort (n=120) and an internal test cohort (n=30), and forty-five patients from another hospital serving as an external test cohort. Radiomics features of uterine fibroids were extracted and selected based on preoperative T2-weighted imaging fat suppression(T2WI-FS)and contrast-enhanced T1WI(CE-T1WI)images, and logistic regression was used to develop the T2WI-FS, CE-T1WI, and combined T2WI-FS + CE-T1WI models, along with the radiomics–clinical model integrating radiomics features with imaging characteristics. The performance and clinical applicability of each model were assessed through receiver operating characteristic (ROC) curve, decision curve analysis (DCA), as well as Network Readiness Index (NRI) and Integrated Discrimination Index (IDI).

**Results:**

The AUC values of the radiomics–clinical model and the T2WI-FS + CE-T1WI model were the highest. In the training cohort, the radiomics–clinical model showed higher AUC values than the T2WI-FS + CE-T1WI model, while in the internal and external testing cohorts, the AUC values of the T2WI-FS + CE-T1WI model were higher than that of the radiomics–clinical model. DCA further demonstrated that these two models achieved the greatest net benefit. NRI and IDI analyses suggested that the T2WI-FS + CE-T1WI model had higher clinical utility.

**Conclusions:**

Both the T2WI-FS + CE-T1WI model and the radiomics–clinical model demonstrate higher predictive value and larger net benefit compared to other models.

## Introduction

Uterine fibroids are the most common benign tumors in the uterus among women (1). Traditional treatments such as surgery, medications, and uterine artery embolization (2, 3) can improve the symptoms of fibroids, but these approaches carry risks such as infection, post-embolization syndrome, and permanent amenorrhea (4). However, the principle of high-intensity focused ultrasound (HIFU) ablation, being a non-invasive approach, involves focusing ultrasound waves on the target tissue under ultrasound monitoring, rapidly raising temperatures to 60°C-100°C, leading to coagulative necrosis of the target tissue (5). At present, High-intensity focused ultrasound (HIFU) is extensively utilized for uterine fibroid treatment (6). Since the treatment outcomes vary among different fibroid patients, accurately predicting the fibroid ablation rate after HIFU treatment preoperatively is crucial for guiding clinical decisions. Magnetic resonance imaging (MRI) has the capability of high-resolution soft tissue imaging, radiation-free characteristics, and multi-sequence imaging, enabling the assessment of the position, size, and vascularity of fibroids. Therefore, it is widely used in preoperative diagnosis and postoperative efficacy evaluation of fibroids (7). However, the analysis of MRI images of uterine fibroids is related to the observer’s experience and is subjective due to the non-quantitative measurement of the features. Radiomics involves converting medical images into multidimensional quantitative data, capturing potential fibroid heterogeneity information. Non-Perfused Volume Ratio (NPVR) is a key parameter for evaluating the efficacy of HIFU treatment, reflecting the ablation status of the uterine fibroid. Therefore, we construct a binary classification model to predict NPVR and, subsequently, the treatment outcome, making it a quantitative, objective, and clinically feasible personalized prediction method for NPVR in HIFU treatment of uterine fibroids (8, 9).

## Methods

### Study population

The study was approved by the Clinical Research Ethics Committee of Yongchuan Hospital Affiliated to Chongqing Medical University (No. 2024LLS005) and the Ethics Committee of the Second Affiliated Hospital of Chongqing Medical University (No.2024-41). Due to the retrospective nature of this study, informed consent was waived. A total of One hundred and ninety-five patients with clinically diagnosed uterine fibroids who were received HIFU treatment between January 2021 and December 2023 were retrospectively analyzed, of which One hundred and fifty patients were collected in our hospital, with ages ranging from 23 to 59 years old and a mean of (45.1 ± 6.4) years old, and Forty-five patients were collected in the another hospital, with ages ranging from 22 to 53 years old and a mean of (38.2 ± 7.2) years old. All patients underwent MRI scans within a week before and after HIFU therapy, and preoperative MRI images were chosen to outline the target area. Inclusion criteria: 1) Patients were not pregnant at the time of examination or had no recent plans for pregnancy. 2) MRI scans were conducted both one week before and after the ablation procedure. 3) Patients did not undergo surgery or any other treatment before the MRI examination. 4) The MR image quality is sufficient for delineating the ROI. Exclusion criteria: 1)Lesions with a major axis length less than 1cm. 2)Poor image quality that prevents ROI delineation and extraction of radiomics features. 3)Other uterine diseases requiring surgical treatment. 4) Prior hormonal therapy that has been instituted or attempted.

### MRI scanning protocol

All patients underwent T2-weighted imaging fat suppression (T2WI-FS), contrast-enhanced T1WI (CE-T1WI) and Diffusion Weighted Imaging (DWI) before and after HIFU treatment. MRI examinations were conducted at our hospital using 3.0T system (Siemens verio dot system), and at another hospital using both 3.0T (Siemens Prisma) and 1.5T (Siemens Avanto) systems. Details of MRI imaging sequences and parameters can be found in **Tables 1**, **2**.

**Table 1:** Main parameters of each sequence of MRI in our hospital.

Sequences	TR(ms)	TE(ms)	Layer thickness (mm)	FOV(mm²)	matrices
T2WI-FS	3430	85	5	240×240	256 x 256
CE-T1WI	3	1	4	325 x 400	260 x 320
DWI	4600	68	5	230 x 230	140 x 140

**Table 2:** Main parameters of each sequence of MRI in another hospital.

Sequences	TR(ms)	TE(ms)	Layer thickness (mm)	FOV (cm)	matrices
T2WI-FS	>2000	60-130	4-6	30-40	≥320×256
CE-T1WI	<5	<2	2-4	30-40	≥320×256
DWI	3000-10000	50-100	4-6	30-40	≥164×140

### Grouping criteria

Measure the longitudinal (D1), anteroposterior (D2), and transverse (D3) diameters of the target uterine fibroid volume and the non-perfused volume (NPV) on contrast-enhanced T1-weighted images before and after HIFU treatment, respectively, and calculate the fibroid volume and NPV using the ellipsoid volume calculation formula *V* = 0.5233 × *D*1 × *D*2 × *D*3. And the non-perfused volume ratio (*NPVR*) = *NPV*/*Fibroid volume* × 100%. Previous research has demonstrated the correlation between NPVR and the efficacy of HIFU treatment, and a NPVR greater than 70% is considered safer (10). Therefore, in this study, a NPVR ≤70% was classified as the low NPVR group, and a NPVR >70% was classified as the high NPVR group.

### Imaging characteristics

The DWI signal intensity of the fibroid is collected (with reference to the uterine muscle layer and skeletal muscle, where signal lower than skeletal muscle is low signal, signal between skeletal muscle and muscle layer is intermediate signal, and signal higher than muscle layer is high signal). The degree of enhancement on T1WI (lower than the myometrium as mild, similar to the myometrium as moderate, and higher than the myometrium as significant) is also collected. Additionally, On sagittal T2-weighted images, measurements are taken for the following parameters: leiomyosarcoma ventral cutaneous distance (shortest distance from the ventral surface of the leiomyosarcoma to the skin of the abdominal wall), leiomyosarcoma dorsal cutaneous distance (longest distance from the dorsal surface of the leiomyosarcoma to the skin of the abdominal wall), and the thickness of the rectus abdominis muscle (measured at the level of the sacrum 2).

### Radiomics feature extraction and feature screening

**Figure 1:**
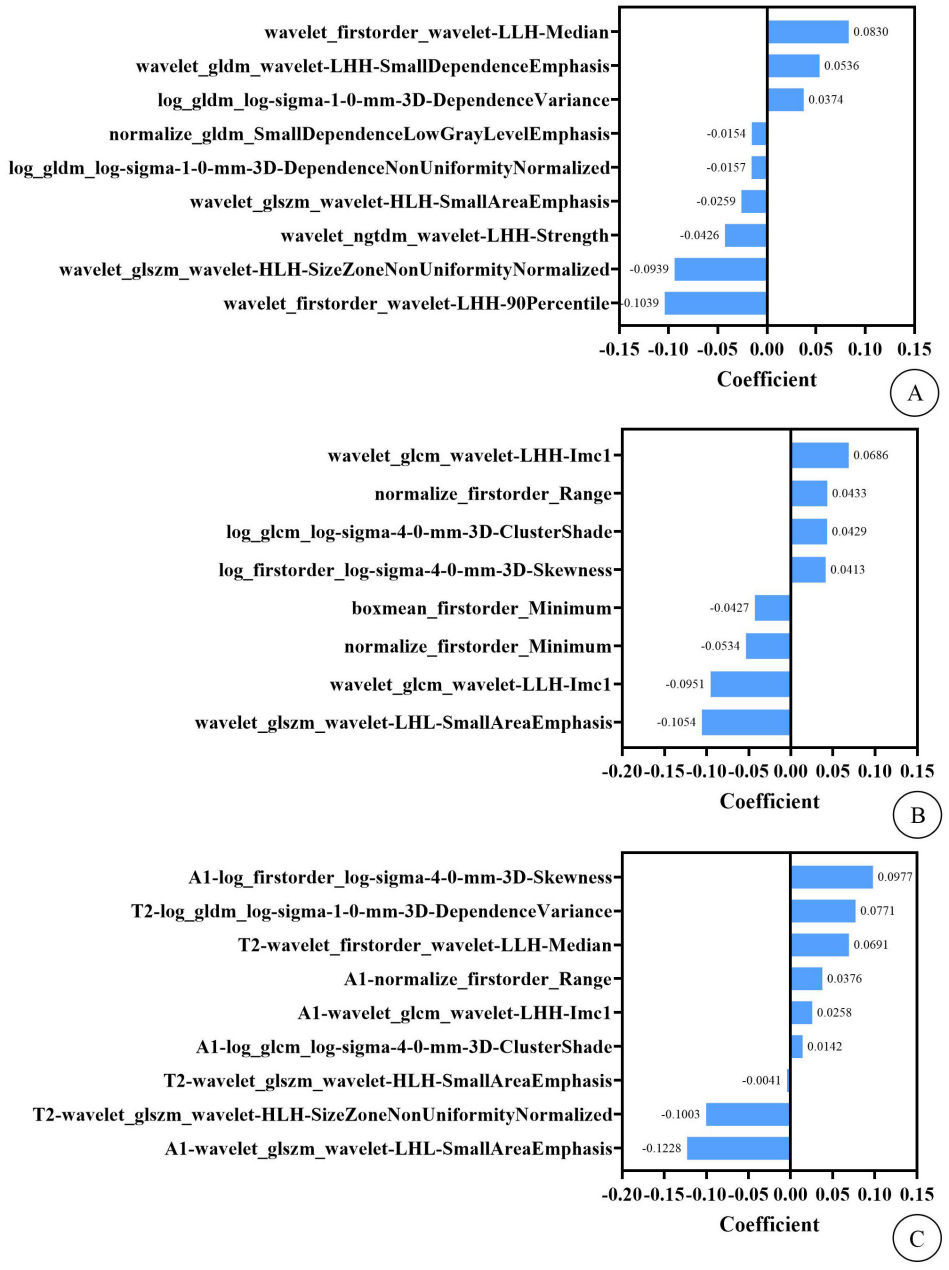
**(A)** shows the imaging features and their weights of T2WI-FS model. **(B)** shows the imaging features and their weights of CE-T1WI model. **(C)** shows the imaging features and their weights of T2WI-FS + CE-T1WI model. In this context, the “T2” and “A1” preceding the imaging features in figure **(C)** represent that these features are extracted from the T2WI-FS and CE-T1WI sequence images, respectively.

A radiologist specializing in pelvic disease diagnosis manually delineated regions of interest (ROIs) along the fibroid edges on preoperative T2WI-FS and contrast-enhanced MRI delayed-phase transverse images to obtain volumes of interest (VOIs). Another experienced radiologist randomly selected 50 cases and manually delineated the contours of the fibroids on the same sequence images. 4460 features were extracted from both sequences in total, including shape-based features, first-order features and texture features. Interclass correlation coefficients (ICC) were analyzed to evaluate the consistency between observers. Features with ICC > 0.75 were retained for feature selection. The Z-score normalization method was applied to preprocess the feature data, and feature selection was conducted using K best, recursive feature elimination, univariate/multivariate logistic regression, and least absolute shrinkage and selection operator. Finally, 9 radiomics features (including 2 first-order features and 7 texture features) and 8 radiomics features (including 4 first-order features and 4 texture features) were selected from T2WI-FS and CE-MRI delayed-phase images, respectively. Subsequently, 4 T2WI-FS features (including 1 first-order feature and 3 texture features) and 5 CE-T1WI features (including 2 first-order features and 3 texture features) were retained through Lasso for the T2WI-FS + CE-T1WI model. The Rad-Score from the two sequences combined with 3 imaging features (fibroid-to-abdominal skin distance, degree of enhancement on T1WI, and DWI signal intensity) were utilized for the radiomics–clinical model, as shown in **Figure 1**.

### Construction and evaluation of predictive models

**Figure 2:**
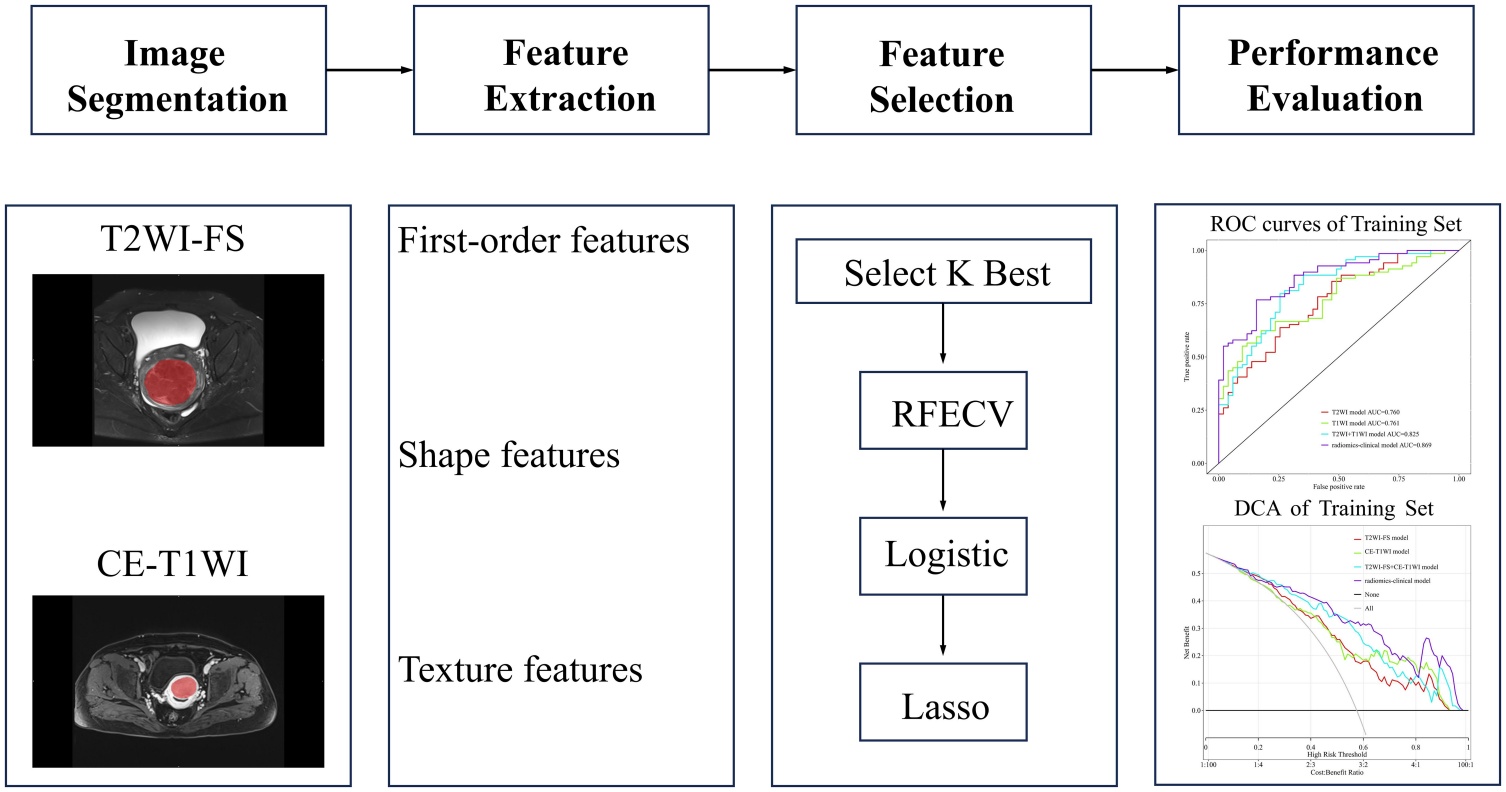
Radiomics workflow diagram. In medical imaging, segmentation is performed to define the fibroid region. Features such as first-order features, shape features, and texture features are extracted from this region. Important features are then selected to construct the predictive model. Finally, the model is evaluated.

A total of 150 patients from our hospital were randomly split into a training cohort (n=120) and an internal test cohort (n=30) at an 8:2 ratio, while the remaining 45 patients from another hospital formed the external test cohort. Using the selected radiomics features and statistically significant imaging features, four prediction models were constructed using logistic regression in the Lianying uAI Research Portal (V730) software: T2WI-FS model, CE-T1WI model, T2WI-FS + CE-T1WI model, and the radiomics–clinical model. The models’ predictive performance was assessed through receiver operating characteristic (ROC) curve analysis, including the area under the curve (AUC), accuracy, specificity, sensitivity, and precision. The radiomics workflow diagram can be seen in **Figure 2**.

### Statistical analysis

We conducted statistical analysis using SPSS 26.0 software, presenting normally distributed continuous data as mean ± standard deviation ( $\bar{x}\pm s$ ), and non-normally distributed continuous data as median (interquartile range).Independent sample t-tests or Wilcoxon rank-sum tests were used for the analysis of continuous data, and chi-square tests or Fisher’s exact tests were used for the analysis of categorical data. Differences were considered statistically significant when p < 0.05. Additionally, the construction of the ROC curves was based on the true positive rate (TPR) and false positive rate (FPR) at different classification thresholds. We employed logistic regression models to perform the classification and calculated the corresponding TPR and FPR to assess the model’s performance.

## Results

### Analysis of imaging data

The differences in age, Leiomyosarcoma ventral cutaneous distance, degree of T1WI enhancement, and DWI signal intensity between the low and high ablation rate groups of one hundred and fifty patients in our hospital were statistically significant; the differences in fibroid volume, the thickness of the rectus abdominis muscle, and leiomyosarcoma dorsal cutaneous distance showed no statistically significant differences (P>0.05) (shown in **Table 3**). The difference in the degree of T1WI enhancement between the high ablation rate group and the low ablation rate group was statistically significant between the forty-five patients in another hospital (P=0.023), and the remaining characteristics were not statistically significant (P>0.05) (shown in **Table 4**).

**Table 3:** Imaging data of patients with uterine fibroids in our hospital.

clusters parameters	High ablation rate group (n=86)	Low ablation rate group (n=64)	P-value
Age (years)	43.9±6.3	46.8±6.1	0.001
Fibroid volume (cm³)	39.90 (14.58, 90.76)	46.43 (15.34, 100.13)	0.885
the thickness of the rectus abdominis muscle(mm)	8.60±3.00	8.66±3.29	0.919
leiomyosarcoma ventral cutaneous distance (mm)	48.03±20.71	59.20±26.32	0.012
leiomyosarcoma dorsal cutaneous distance (mm)	97.11±23.17	102.77±24.05	0.148
DWI signal (I00%)			0.039
low signal	45 (52.3%)	21 (32.8%)	
isosignal	18 (20.9%)	23 (35.9%)	
high signal	23 (26.7%)	20 (31.2%)	
Degree of T1WI enhancement (100%)			0.002
mild enhancement	48 (55.8%)	18 (28.1%)	
moderate enhancement	18 (20.9%)	16 (25.0%)	
significant enhancement	20 (23.3%)	30 (46.9%)	

**Table 4:** Imaging data of patients with uterine fibroids in another hospital.

clusters parameters	High ablation rate group (n=41)	Low ablation rate group (n=4)	P-value
Age (years)	37.9±7.1	41.0±8.5	0.417
Fibroid volume (cm³)	81.15 (52.13, 132.29)	73.49 (45.75, 107.15)	0.632
the thickness of the rectus abdominis muscle(mm)	6.1±2.6	7.3±2.5	0.362
leiomyosarcoma ventral cutaneous distance (mm)	46.10 (36.70, 67.50)	46.65 (23.80, 82.85)	0.780
leiomyosarcoma dorsal cutaneous distance (mm)	103.3±18.6	100.7±29.3	0.803
DWI signal (I00%)			0.533
low signal	15 (36.6%)	2 (50.0%)	
isosignal	13 (31.7%)	2 (50.0%)	
high signal	13 (31.7%)	0 (0.0%)	
Degree of T1WI enhancement (100%)			0.023
mild enhancement	20 (48.8%)	0 (0.0%)	
moderate enhancement	7 (17.1%)	3 (75.0%)	
significant enhancement	14 (34.1%)	1 (25.0%)	

### Model evaluation

**Figure 3:**
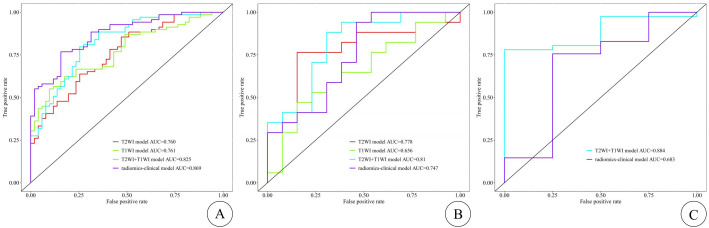
The ROC curves of the training, internal test, and external test cohorts are shown in **(A–C)**, respectively, with the T2WI-FS model represented by the red curve, the CE-T1WI model by the green curve, the T2WI-FS + CE-T1WI model by the blue curve, and the radiomics–clinical model by the purple curve. The X-axis represents 1-specificity (false positive rate), and the Y-axis represents sensitivity (true positive rate). The ROC curve divides the black box into two parts, with the area below representing the AUC. The diagonal line (non-informative curve) indicates the result of random guessing, meaning the model has no discriminative ability, with the true positive rate and false positive rate being equal.

Each model’s performance was assessed using metrics including the area under the curve (AUC) of the receiver operating characteristic (ROC) curve, sensitivity, specificity, accuracy, and F1 score (shown in **Table 5**). Among these four models, models C and D achieved the highest AUC values. In the training cohort, model D outperformed model C in terms of AUC, while in both the internal and external testing cohorts, model C exhibited higher AUC values than model D (shown in **Figure 3**). To provide a comprehensive assessment of model performance, In 2008,Pencina et al. (11) proposed the Network Readiness Index (NRI) and Integrated Discrimination Index (IDI) to examine whether the diagnostic accuracy of a certain indicator relative to another indicator improves and to evaluate overall model improvement. We compared each of the four models two by two, and found that for Models C in both the training and validation sets, compared to Models A, B, and D, the Net Reclassification Improvement (NRI) values were all greater than 0 (0.22, 0.21, 0.06), Models C in the training sets, compared to Models A, B, and D, the Integrated Discrimination Improvement (IDI) values were all greater than 0 (0.22, 0.17, 0.008), indicating that Model C had the highest predictive accuracy and its predictive model was improved positively. The NRI(0.20) and IDI (0.20)of Model C compared with the external validation set of Model D are greater than 0, indicating that Model C has higher prediction accuracy and higher clinical utility.

**Table 5:** Indicators for the assessment of models.

Model		AUC	(level of) sensitivity	specificity	accuracy	F1 rating
A	training cohort internal test cohort	0.760 0.778	0.696 0.765	0.627 0.692	0.667 0.733	0.706 0.765
B	training cohort internal test cohort	0.761 0.656	0.657 0.647	0.680 0.615	0.667 0.633	0.697 0.667
C	training cohort internal test cohort external test cohort	0.825 0.810 0.884	0.886 0.882 0.778	0.660 0.615 0.750	0.792 0.767 0.778	0.832 0.811 0.865
D	training cohort internal test cohort External test cohort	0.869 0.747 0.683	0.623 0.647 0.585	0.863 0.615 0.750	0.725 0.633 0.600	0.723 0.667 0.727

A represents the T2WI-FS model, B represents the CE-T1WI model, C represents the T2WI-FS + CE-T1WI model, and D represents the radiomics–clinical model.

### Clinical application

**Figure 4:**
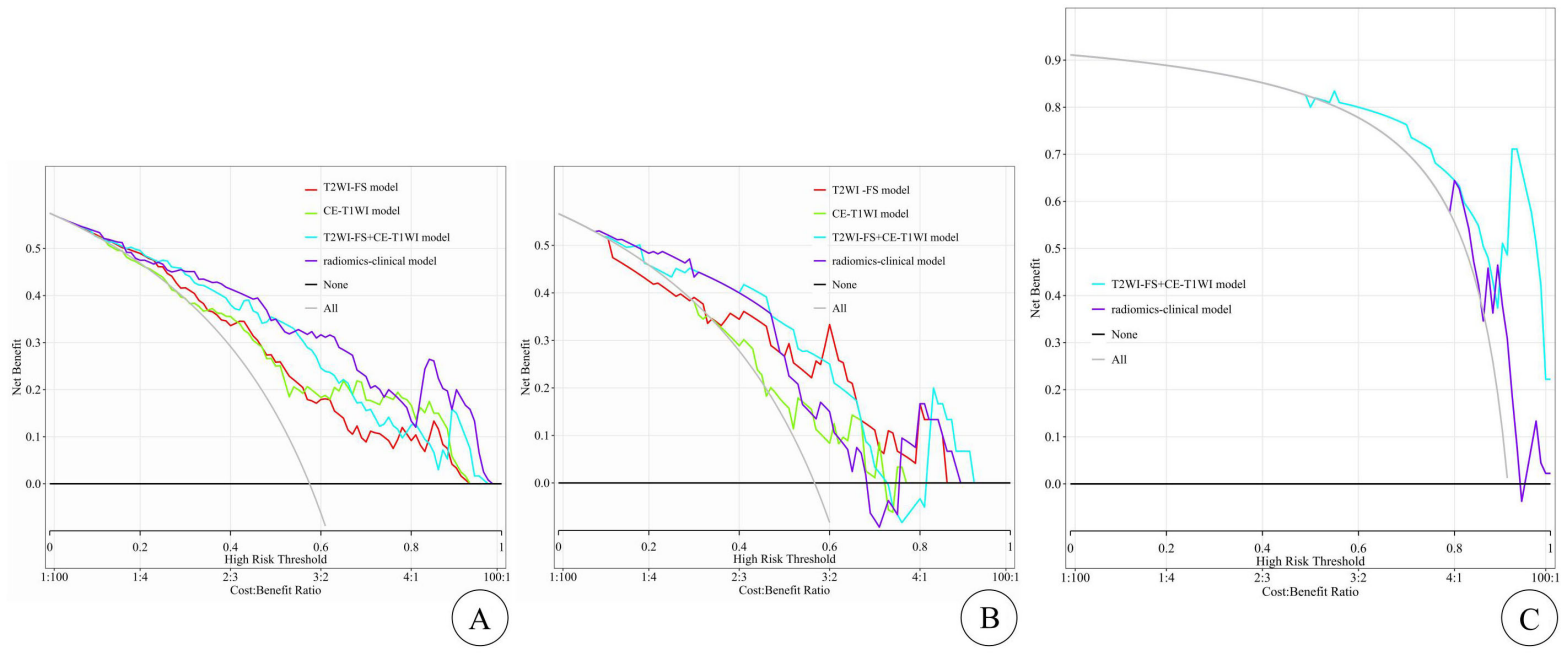
The DCA curves for clinical application assessment show the net benefit on the y-axis and threshold probability on the x-axis, with **(A)** for the training cohort, **(B)** for the internal test cohort, and **(C)** for the external test cohort. The T2WI-FS model is represented by the red line, the CE-T1WI model by the green line, the T2WI-FS+CE-T1WI model by the blue line, and the radiomics-clinical model by the purple line. The gray line and black line represent the “all treatment” and “no treatment” strategies, respectively.

The DCA curves for assessing clinical applications are shown in **Figure 4**, depicting the results for each model. Compared to other models, the T2WI-FS + CE-T1WI model and radiomics–clinical model show a higher net benefit within the threshold probability range (shown in **Figure 4**).

## Discussion

### Comparison of related studies

Radiomics can extract valuable information from medical images that are imperceptible to the naked eye and transform it into quantitative features, thus objectively supplementing valuable information (12, 13). Numerous researchers have confirmed the value of radiomics in tumor diagnosis (14), staging (15) and predicting treatment efficacy. Currently, Some researchers have achieved promising results by applying radiomics to assess the effectiveness of HIFU treatment for uterine fibroids (16, 17). For example, Jiang et al. (18) and Qin et al. (19) extracted radiomic features from contrast-enhanced MRI and T2WI-FS single-sequence images, respectively, to build predictive models, both demonstrating good predictive performance but lacking external validation. Zhou et al. also demonstrated the excellent predictive performance of a radiomics-clinical prediction model based on T2WI single-sequence images (8). Li et al. (20) constructed superior radiomics models using LightGBM (Light Gradient Boosting Machine) based on T2WI and CE-T1WI, with AUC values of 0.872 and 0.848, respectively, which did not combine the two sequences in the model. Zheng et al. (21) built four joint models based on T2WI and DWI (Diffusion-Weighted Imaging) sequences using four machine learning algorithms, showing that the SVM model had the best predictive performance. However, this model did not use the commonly used CE-T1WI sequence, and the study was single-center and lacked generalization to test data. In this study, the radiomics joint model based on T2WI-FS and CE-T1WI sequences can more comprehensively extract high-throughput features of uterine fibroids than models based on single sequences, consistent with the findings of Zheng et al. (22). Moreover, through external validation and generalization of the test set data, this study enhances the model’s reliability, enabling it to predict HIFU efficacy preoperatively and assist patients in formulating appropriate treatment plans.

### The analysis of imaging features research results

In this study, we found that age, leiomyosarcoma ventral cutaneous distance, degree of T1WI enhancement, and DWI signal intensity were the influencing factors of ablation rate in One hundred and fifty patients in our hospital, whereas only the degree of T1WI enhancement was significant in forty-five patients in another hospital, which may be due to the smaller sample size. HIFU is a technique that results in coagulative necrosis of the target tissues by emitting focused ultrasound waves on the target tissues, Ultrasound waves are susceptible to the influence of non-target tissues, such as refraction, reflection, and absorption, as they pass through the acoustic pathway, leading to energy attenuation. Therefore, there is a negative correlation between the ventral skin distance of leiomyosarcoma and the HIFU ablation rate of leiomyosarcoma, meaning that the shorter the distance from leiomyosarcoma ventrally to the skin, the less energy attenuation occurs, resulting in a better ablation effect. This finding is consistent with the results of studies by Song et al. (23) and Gong (24) et al. The enhancement degree on T1-weighted imaging (T1WI) reflects the blood supply situation of the leiomyosarcoma tissue, and the higher the degree of T1WI enhancement, the richer the leiomyosarcoma’s blood perfusion. In this study, there was a negative correlation between the enhancement degree on contrast-enhanced T1-weighted imaging (CE-T1WI) and the ablation rate, consistent with previous research results (25). This is because a significantly enhanced leiomyosarcoma indicates a richer blood supply, and blood flow may lead to the loss of ultrasound energy, resulting in reduced energy accumulation in the target tissue and increased difficulty in ablation. Diffusion-Weighted Imaging (DWI) provides information about the structure of leiomyosarcoma tissue, cellular composition, and microcirculation of capillaries (7). A previous study (26) showed that DWI can reflect the diffusion degree of water molecules within leiomyosarcoma tissue. In this study, leiomyosarcomas with high signal intensity on DWI showed poorer ablation rates. This might be attributed to the high extracellular interstitial water levels and/or increased blood volume in leiomyosarcomas with high signal intensity on DWI, which hinder energy deposition. This finding is in agreement with the previous study results (27, 28). The ventral skin distance of leiomyosarcoma, enhancement pattern on T1-weighted imaging (T1WI), and signal intensity on DWI are crucial indicators for assessing energy attenuation and deposition during High-Intensity Focused Ultrasound (HIFU) treatment. They play a pivotal role in predicting the ablation rate. Therefore, in this study, they were utilized to establish a radiomics-imaging fusion model.

## Limitations

The limitations of this study are as follows: (1) The limited amount of data in this study might have led to the lack of statistical significance in the Delong analysis of the internal and external validation sets. Further inclusion of more data is warranted. (2) Errors may have been introduced during the manual delineation of the tumor boundaries. (3) this study is retrospective and there is a selection bias in the selection of patients.

## Conclusion

This study demonstrates that both the combined model constructed based on T2WI-FS and CE-MRI radiomic features and the fusion model based on combined radiomic and imaging features can effectively predict the short-term efficacy of HIFU treatment before surgery. This will assist clinicians in better selecting patients who will benefit from HIFU and in formulating precise treatment plans. Future research will involve multi-center, large-sample studies and the development of more comprehensive predictive models based on multiparametric MRI and laboratory parameters to enhance the model’s predictive capabilities and validate its effectiveness, thereby contributing to the advancement of precision medicine.

## Data Availability

The datasets presented in this article are not readily available because the data analyzed in this study is subject to the following licenses/restrictions: The datasets for this article are not publicly available as it is private data that belongs to The Affiliated Yongchuan Hospital of Chongqing Medical University. Requests to access the datasets should be directed to corresponding author. Requests to access the datasets should be directed to Quan Yang, 2644738456@qq.com.
